# An anti-CD6 monoclonal antibody (itolizumab) reduces circulating IL-6 in severe COVID-19 elderly patients

**DOI:** 10.1186/s12979-020-00207-8

**Published:** 2020-11-14

**Authors:** Danay Saavedra, Ana Laura Añé-Kourí, Naivy Sánchez, Lázaro Manuel Filgueira, Julio Betancourt, Carlos Herrera, Leniel Manso, Elibet Chávez, Armando Caballero, Carlos Hidalgo, Geydi Lorenzo, Meylan Cepeda, Carmen Valenzuela, Mayra Ramos, Kalet León, Zaima Mazorra, Tania Crombet

**Affiliations:** 1 Department of Clinical Immunology. Center of Molecular Immunology, 216 St, Corner 15, PO Box 16040, Havana, Atabey Cuba; 2 Biochemical Department. ICBP “Victoria de Girón”, Calle 146 # 3102, La Habana, Playa Cuba; 3 Manuel Piti Fajardo Universitary Hospital. Ciudad Escolar Abel Santamaría, U/M 9958, Santa Clara, Villa Clara Cuba; 4 Arnaldo Milian Castro Universitary Hospital, Arnaldo Milián Castro St, Santa Clara, Villa Clara Cuba; 5 Cardiocentro “Ernesto Che Guevara”, Cuba St N° 610, Santa Clara, Villa Clara Cuba

**Keywords:** COVID-19, IL-6, Itolizumab, Cytokine release syndrome

## Abstract

**Background:**

Since the COVID-19 outbreak an unprecedented challenge for healthcare systems around the world has been placed. In Cuba, the first case of COVID-19 was reported on March 11. Elderly with multiple comorbidities have been the most risky population. Although most patients present a mild to moderate disease, some have developed severe symptoms. One of the possible mechanisms underlying rapid disease progression is a cytokine storm, in which interleukin (IL) -6 seems to be a major mediator. Itolizumab is a humanized recombinant anti-CD6 monoclonal antibody (MAb), with the ability of reducing serum interferon gamma (INF-γ), tumour necrosis factor alpha (TNFα) and IL-6. Based on these previous results in patients with psoriasis and rheumatoid arthritis, an expanded access clinical trial was approved by the Cuban regulatory agency for COVID-19 critically, severely and moderately ill patients.

**Results:**

We show here a short kinetic of IL-6 serum concentration in the first 24 COVID-19 patients treated with itolizumab. Most of patients were elderly with multiple comorbidities. We found that with one itolizumab dose, the circulating IL-6 decreased in critically and severely ill patients, whereas in moderately ill patients the values didn’t rise as compared to their low baseline levels.

**Conclusion:**

These findings suggest that itolizumab could be an attractive therapeutic option to decrease the negative outcome of the cytokine storm in COVID-19 patients.

**Trial registration:**

CECMED IIC RD-EC 179, RPCEC00000311. Registered 4 May 2020 - Retrospectively registered, http://rpcec.sld.cu/ensayos/RPCEC00000311-Sp or http://rpcec.sld.cu/trials/RPCEC00000311-En

## Background

The Severe Acute Respiratory Syndrome Coronavirus 2 (SARS-CoV-2) has caused a recent outbreak of Coronavirus Disease (COVID-19) [1, 2]. The disease started in Wuhan, China in December 2019 and has rapidly spread throughout the world [1]. To date, the disease has already affected more than five million globally, with a 6.48% of mortality. In Cuba, the first case of COVID-19 was reported on March 11, 2020. Since then, the number of positive cases has risen to 1947 cases, according to government data on May 25, 2020. Notably, the epidemiological curve is decreasing, moving forward daily reduction of the cases.

Although most cases are mild to moderate, some patients developed severe symptoms characterized by respiratory dysfunction and/or multiple organ failure that causes the death in most cases [3]. Previous studies have revealed that patients with old age and comorbidities such as hypertension and diabetes are more likely to be aggravated [4, 5].

Altered immune competence with increasing age, defined as immunosenescence and the state of chronic, sterile, low-grade inflammation known as inflammaging, characterized the immune system in the elderly. Both processes together are suggested as the origin of most of the comorbidities of the elderly and its susceptibility to suffer cancer, chronic inflammatory diseases and new infections [6, 7]. The age-associated diseases together with the aging of the structure and function of the lungs increase the likelihood of serious progression of respiratory viral infection [8].

It has been suggested that one of the possible mechanisms underlying rapid disease progression is a cytokine storm, in which IL-6 seems to be a major mediator [9, 10]. Previous retrospective studies indicated that an elevated level of interleukin-6 (IL-6) was associated with a high case fatality of COVID-19 infection [1]. Based on emerging information treating SARS-CoV- 2-infected patients, modulating or inhibiting the IL-6 signaling pathway to mitigate the inflammatory response related to COVID-19 is an attractive idea. At present, several clinical trials are under way to evaluate the safety and efficacy of IL-6 inhibitors, with various protocols and comparators [11]. Preliminary results in a limited number of patients suggested the use of tocilizumab as promising therapeutic agent for severe and critical SARS-CoV-2 infections [12]. Nevertheless, this therapy should be used with caution taking into account the increase in serious bacterial infections reported in tocilizumab-treated patients [13].

Itolizumab is a humanized recombinant anti-CD6 monoclonal antibody (MAb) of immunoglobulin G1 (IgG1) isotype which binds to domain 1 of human CD6. This MAb was developed at the Center of Molecular Immunology (CIM, Havana, Cuba) and has demonstrated to be effective and safe in randomized clinical trials performed in psoriatic patients [14]. Itolizumab modulates T-lymphocytes activation and proliferation induced by CD6-costimulation. The regulation of downstream pathways such as pMAPK, pSTAT3 and pAKT further results in reduction of INF-γ, TNFα and IL-6 both in vitro and in vivo [15, 16]. Itolizumab is not a T cell depleting agent and whenever depletion occurs, it is transient in nature [17]. Recently, it was suggested the use of this MAb to treat the cytokine release syndrome detected in severe COVID-19 patients (Rodriguez PC et al., manuscript in preparation). Based on the potential use of itolizumab in this disease an expanded access trial was approved by Cuban regulatory agency (CECMED). The trial is recruiting patients with critical and severe illness; also, moderately ill patients with very high risk of developing severe symptoms.

In this paper, we show the results of the first 24 patients treated with itolizumab. The immense majority were elderly with multiple comorbidities. For the first time, it is reported that this antibody is able to decrease circulating IL-6 levels in patients with critical and severe COVID-19.

## Results

### Characteristics of COVID-19 patients

Twenty-four laboratory-confirmed patients, 6 males (25%) and 18 females (75%) were treated with itolizumab monoclonal antibody. The patients were categorized into three groups regarding the severity of illness: moderately ill (elderly with various symptoms including polypnea and O2 requirement), *n* = 11; severely ill (SpO2 ≤ 93% while breathing room air, requiring additional O2 supply), *n* = 7 and critically ill (patients with respiratory failure, requiring mechanical ventilation among other conditions), *n* = 6. Only two patients (8.3%) were younger than 65 years old. The average of age was 79.83 years old (Table 1).

**Table 1:** Demographics and baseline characteristics of moderately, severely and critically ill COVID-19 patients

	Moderately ill Patients	Severely ill patients	Critically ill patients	Total
	(***n*** = 11)	(***n*** = 7)	(***n*** = 6)	
**Age (mean), years**	80	85.14	73.33	79.83
**Sex (%)**				
Male	3 (12.5%)	0	3 (12.5%)	6 (25%)
Female	8 (33.33%)	7 (29.16%)	3 (12.5%)	18 (75%)
**Most frequent comorbidities (%)**				
Hypertension	8 (33.33%)	3 (12.5%)	3 (12.5%)	14 (58.33%)
Diabetes mellitus	4 (16.66%)	1 (4.16%)	3 (12.5%)	8 (33.33%)
Cardiovascular diseases	2 (8.33%)	2 (8.33%)	3 (12.5%)	7 (29.16%)
COPD	1 (4.16%)	1 (4.16%)	0	2 (8.33%)
Cancer	0	1 (4.16%) NSCLC	0	1 (4.16%)

Abbreviations: *COPD* Chronic obstructive pulmonary disease; *NSCLC* Non-small cell lung cancer

Most of the patients presented several comorbidities at the moment of SARS-CoV-2 diagnosis predominantly hypertension, diabetes mellitus and cardiovascular diseases (Table 1).

### Laboratory findings

Neutrophil number had significant differences among the three groups, especially between moderately ill and critically ill patients (4.462 vs 9.57; *p* = 0.013, ANOVA, Tukey’s multiple comparison test) and severely ill and critically ill patients (4.77 vs 9.57; *p* = 0.032, ANOVA, Tukey’s multiple comparison test). Critically and severely ill patients had higher neutrophil-to-lymphocyte ratio (NLR) than moderately ill (10.82 and 6.38 vs 3.8; *p* = 0.06, Kruskall-Wallis test), although no statistical significance was achieved. The rest of the hematological and biochemical parameters evaluated, were not different between the groups (Table 2).

**Table 2:** Laboratory parameters in moderately, severely and critically ill COVID-19 patients

Baseline parameters	Moderately ill Patients	Severely ill patients	Critically ill patients	All patients	***p*** value
	(***n*** = 11)	(***n*** = 7)	(***n*** = 6)		
Hemoglobin (g/l)	112.4 ± 16.63	104 ± 22.35	90.17 ± 37.73	102.6 ± 27.26	0.744 ANOVA
White blood cell (× 10^9^/L)	6.925 ± 2.842	7.92 ± 2.366	11.08 ± 4.304	8.5 ± 3.599	0.090 K-W
Neutrophil (× 10^9^/L)	4.462 ± 2.055	4.777 ± 3.417	9.537 ± 4.154	5.882 ± 3.712	**0.012** ANOVA
Lymphocyte (× 10^9^/L)	1.571 ± 0.6184	1.634 ± 0.689	1.059 ± 0.5516	1.44 ± 0.6363	0.227 ANOVA
NLR	3.825 ± 3.317	6.389 ± 9.717	10.82 ± 6.083	6.433 ± 6.501	0.065 K-W
Platelet (× 10^9^/L)	198.5 ± 41.79	231.2 ± 32.69	311 ± 156.9	224 ± 88.83	0.413 ANOVA
PLR	171.8 ± 154.5	243.2 ± 251.9	226.9 ± 223.4	227.8 ± 195.8	0.208 K-W
Triglycerides (mmol/L)	1.023 ± 0.2329	1.197 ± 0.2871	1.962 ± 1.081	1.408 ± 0.7893	0.080 ANOVA
ALT (U/L)	18.86 ± 13.63	18 ± 15.75	36.67 ± 22.54	24.94 ± 18.84	0.168 ANOVA
D-Dimer (mg/l)	0.92 ± 0.6479	2.07 ± 1.669	1.773 ± 0.7467	1.396 ± 0.924	0.744 ANOVA

Abbreviations: *NLR* Neutrophil-to-lymphocyte ratio; *PLR* Platelet-to-lymphocyte ratio; *ALT* Alanine aminotransferase; *ANOVA* Analysis of variance; *K-W* Kruskall-Wallis

### Serum cytokines

**Fig. 1:**
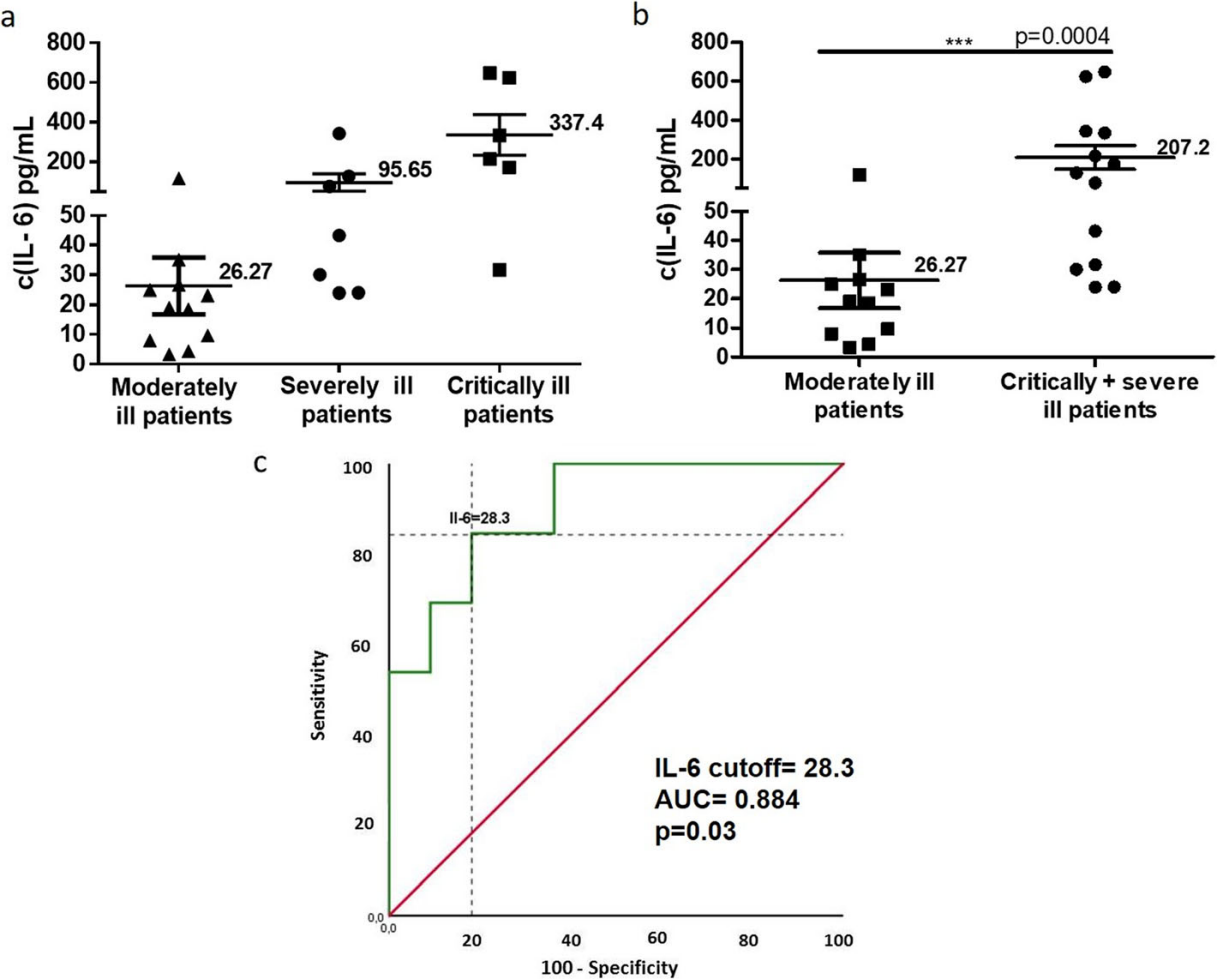
IL-6 concentration in the sera of COVID-19 patients **a**) Mean of IL-6 levels in the three groups of COVID-19 patients. **b** The values are significantly higher in the group of critically and severely ill patients than in moderately ill patients. c) ROC curves of IL-6 predictive value for the severity of COVID-19. The asterisks indicate statistically significant differences among the groups (*p* < 0.05) (*) using Mann Whitney test. ROC: receiver operator characteristic; AUC: area under curve

There were no differences in IL-1 and TNFα serum concentration among the groups (data not shown). Actually, the majority of patients had no detectable levels of these inflammatory cytokines. In contrast, IL-6 was overexpressed. IL-6 levels increased with the progression of severity (Fig. 1a). The serum concentration in critically ill and severely ill patients was significantly higher than in moderately ill patients (Fig. 1b). The mean serum IL-6 was 337.4 pg/mL for critically ill patients; 95.65 pg/mL, for severely ill and 26.27 pg/mL for moderately ill patients.

The baseline IL-6 levels were related to the severity of illness when applying a receiver operator characteristic (ROC) curve (*p* = 0.003). The area under curve (AUC) of IL-6 was 0.884, the sensitivity 84.6%, the specificity 81.8% and the cutoff value of IL-6 selected was 28.3 pg/ml (Fig. 1c).

### Itolizumab reduces IL-6 in critically and severely ill patients and stabilizes its levels in moderately ill patients

**Fig. 2:**
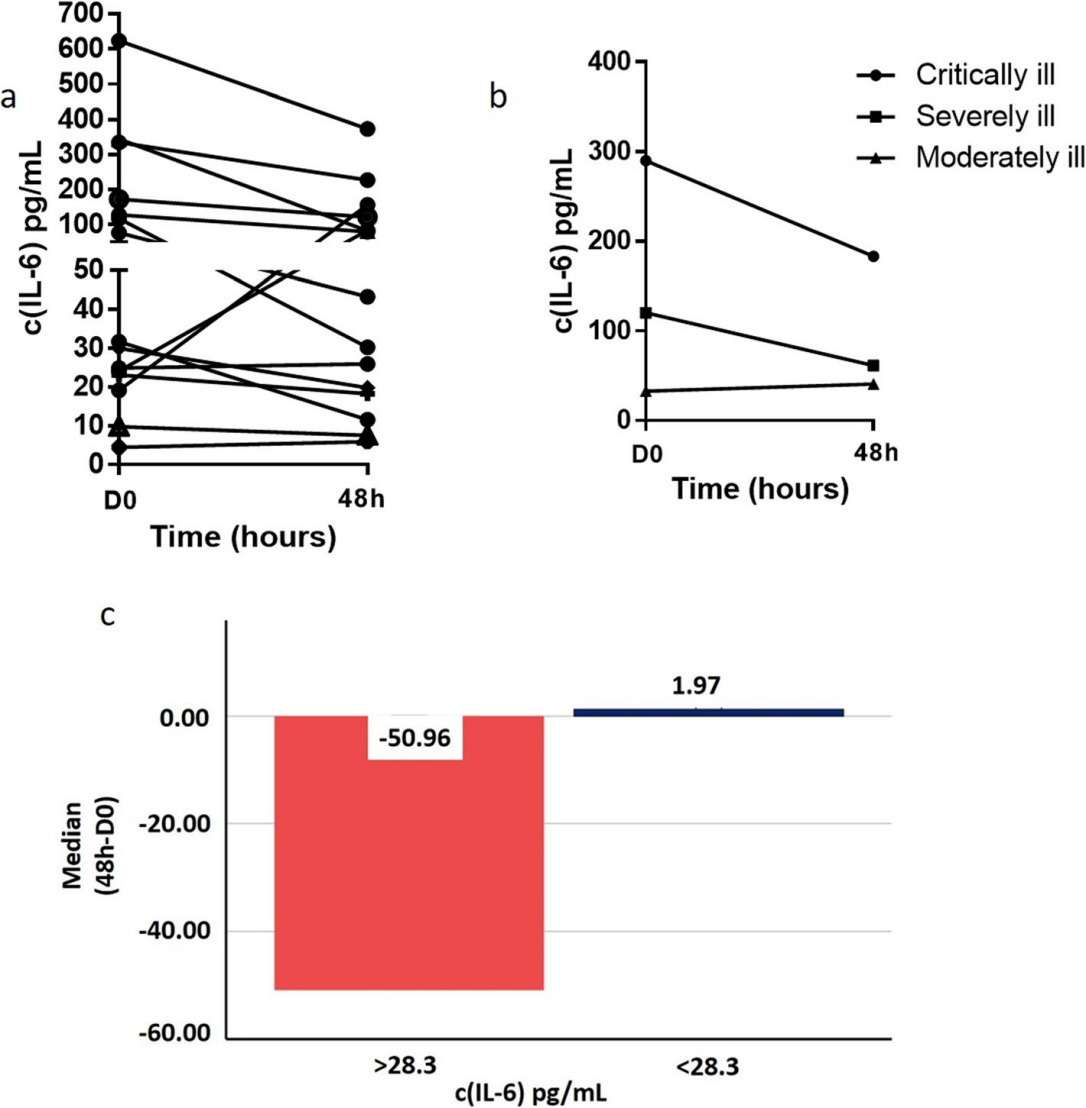
IL-6 serum concentration in COVID-19 patients before and 48 h after the treatment with itolizumab. **a** Individual behavior of IL-6 values in the patients. **b** Kinetic of the mean of IL-6 levels in the three groups of patients. **c** Magnitude of change of IL-6 concentration 48 h after the administration of the first itolizumab dose in COVID 19 patients with pre-treatment levels higher than 28.3 pg/mL and lower than 28.3 pg/mL. D0: Before treatment with itolizumab; 48 h: 48 h after the treatment

Serum IL-6 was measured in patients treated with itolizumab the day of the first administration and 48 h later (*n* = 15). The majority of patients (86.66%) decreased or did not increase its IL-6 values in this period. Only two patients (13.34%) increased the serum IL-6 levels after the treatment (Fig. 2a). The mean values of IL-6 in the critical group reduced from 290.2 pg/mL to 183.1 pg/mL, 48 h after the treatment. Similarly, in severely ill patients the values dropped twice, until 61.4 pg/ml. In the case of moderately ill patients, the circulating IL-6 levels were similar to the pre-treatment values (Fig. 2b).

The cutoff selected by ROC curve to stablish the association between baseline IL-6 concentration and severity of illness was 28.3 pg/mL (Fig. 1c). Remarkably, all patients with pre-treatment circulating IL-6 levels above 28.3 pg/mL, significantly decreased IL-6 concentration with one dose of itolizumab, measured 48 h after the administration. The magnitude of change of IL-6 among the patients with concentrations above the cutoff has a median of reduction of 50 pg/mL (*p* = 0.005, Wilcoxon test, Fig. 2c). However, the median of change in IL-6 concentration among the patients with baseline levels below 28.3 pg/mL, was 1.27 pg/mL (*p* = 0.068, Wilcoxon test).

## Discussion

Since the COVID-19 outbreak, an unprecedented challenge for healthcare systems around the world has been placed [18]. According to the World Health Organization, elderly with multiple comorbidities have the highest risk of developing a severe illness [19]. The immune system of elderly is characterized by immunosenescence and inflammaging. These age-related processes are always put forward to explain the susceptibility of older adults to new infections and chronic diseases such as cardiovascular diseases, diabetes and cancer [20]. This report describes by the first time, the effect of an anti-CD6 monoclonal antibody (itolizumab) decreasing circulating IL-6 in critically and severely ill elderly COVID-19 patients.

Cytokine release syndrome (CRS) was found to be the major cause of morbidity in patients infected with SARSCoV and MERS-CoV, previous coronavirus infections producing severe respiratory illness in humans [21]. Similar to these findings, severe COVID-19 has been described in some patients, accompanied by a similar damaging immune reaction. This severe manifestation is characterized by pronounced infiltration of macrophages and monocytes into alveoli and a pro-inflammatory response with increase of inflammatory cytokines mainly IL-6, IL-1, IFNγ and TNFα [22, 23]. Different papers have demonstrated a correlation between high serum IL-6 levels and adverse clinical outcomes in patients with COVID-19 [1, 24]. Similarly, an extremely high concentration of IL-6 is a driving force of cytokine storm, which may cause multiple organ dysfunctions in critically ill patients [25]. The data reported on IL-6 levels during the COVID-19 outbreak show an evident heterogeneity. A systematic review and meta-analysis of seven studies, performed by Aziz and colleagues reported the mean of serum IL-6 in 56.8 pg/mL (41.4–72.3 pg/mL) for severe COVID-19 group [26], while both our group and Xu et al. found higher IL-6 values for severe and critical COVID-19 patients [12]. Different methods, different specimens (serum or plasma) and the different classification of disease severity could explain this heterogeneity. However, the highest concentrations of IL-6 are always in association with severe disease [3, 5, 27, 28], which is in line with our results.

In addition, high values of neutrophils characterized our critically and severely ill patients. Besides, as it has been previously reported [4, 23, 28], a higher number of neutrophils and a lower number of lymphocytes, conducting to the increase of NLR, were found in the critical and severe groups compared to the moderate group of patients.

Modulation of the CRS and the severe inflammatory state in patients with COVID-19, is a very important strategy to limit the severity of COVID-19 pulmonary and systemic complications. This approach could successfully reduce the needs for intensive care support and mechanical ventilation, and eventually decrease mortality [18]. The use of immunosuppressants, specifically the IL-6 inhibitors, in the management of SARS-CoV-2-associated pneumonia is an attractive option to overcome the inflammation and CRS in the lungs. In this sense, the anti-human IL-6 receptor monoclonal antibody tocilizumab, has been used in a limited number of COVID-19 patients with promising results. Predictive biomarkers of clinical succeed using this MAb are currently investigated since some patients are not benefited [29]. In addition, the use of this antibody should be managed with caution because the high risk of bacterial infection reported among its main adverse events [13].

In our center, we have developed a first- in-class antagonistic MAb that selectively targets the CD6-ALCAM pathway, reducing the activation, proliferation and differentiation of T cells into pathogenic effector T cells and leading to a decrease of pro-inflammatory cytokine production (IL-6, TNF-α and INFγ). At the same time, itolizumab preserves the regulatory function of Treg cells and reduces T cell trafficking and infiltration at the inflammation sites [15]. The safety and efficacy of itolizumab in the treatment of patients with rheumatoid arthritis and severe chronic plaque psoriasis have been demonstrated in several clinical studies [30, 31]. According to this, itolizumab become an alternative immunomodulatory intervention that seeks to control the T cell hyperactivated status in order to prevent such CRS. In line with this rationale, an expanded access clinical trial is recruiting patients with critical and severe illness and moderately ill COVID-19 patients with very high risk of developing severe symptoms.

Our patients treated with itolizumab decreased the circulating IL-6 levels 48 h after the administration, especially in the case of critically and severely ill patients. All the patients with IL-6 baseline levels above 28.3 pg/mL, the cutoff selected by ROC curve to classify the patients with severe disease, significantly decreased the IL-6 concentration.

In the case of moderately ill patients, the baseline serum values didn’t increase 48 h later. The immunomodulatory therapy was administered shortly after the contagion. In addition to the advance age, all moderately ill patients suffered with comorbidities, which are both risk factors associated with severity and fatal outcome in the course of COVID-19 [32, 33]. Therefore, we suggest that the stabilization of the IL-6 levels over low values was due to the treatment with itolizumab.

Recent studies showed that IL-6 and granulocyte-macrophage colony-stimulating factor (GM-CSF) could be secreted by active pathogenic T cells upon SARS-CoV-2, also CD14 + CD16+ inflammatory monocytes activated by GM-CSF could secrete IL-6 and other inflammatory factors [12, 34]. Previous work demonstrated the in vitro capacity of itolizumab inhibiting the induced proliferation of peripheral blood mononuclear cells (PBMC) in the presence of ALCAM and reducing its IFN-γ, IL-6 and TNF-α production [15]. Additionally, a significant decrease in the levels of IL-6, TNF-α and IFN-γ was found in patients with rheumatoid arthritis and psoriasis treated with itolizumab [16, 17]. In this study, the serum TNF-α concentration were undetectable in the majority of the patients, regardless of the severity of illness. Other researchers have found low levels of this cytokine in COVID-19 patients [23, 28]. Interestingly, it has been reported that levels of TNF-α and IL-1β, and some chemotactic cytokines (IL-8 and MCP-1) rise early in COVID-19 hypercytokinemia, facilitating a subsequent sustained increase in IL-6 [35].

This work has some limitations regarding to the small number of patients, the short follow up of IL-6 levels in serum and the lack of the evaluation of samples from untreated patients as controls. This will be addressed in the forthcoming studies. Nevertheless, it is the first report highlighting the ability of itolizumab reducing circulating IL-6 in severe COVID-19 patients. The effect of itolizumab in the clinical outcome of treated patients remains to be analyzed.

## Conclusions

The results from this work show that one dose of itolizumab reduced the baseline serum levels of IL-6 in critically and severely ill COVID-19 patients as well as stabilized the baseline low levels in moderately ill elderly COVID-19 patients. Due to the very safe profile of itolizumab, this MAb could be an appealing option to decrease the negative outcome of the cytokine storm in COVID-19 patients.

## Methods

### Patients and treatment

Twenty-four patients with diagnosis of COVID-19 classified as critically ill, severely ill and moderately ill, were included in an expanded access trial to receive itolizumab, an anti -CD6 monoclonal antibody in addition to standard treatment. The diagnosis of disease severity was defined as following:

Critically ill patients were diagnosed if any of the following conditions were met: respiratory failure, requiring mechanical ventilation, septic shock, and or multiple organ failure. Severely ill patients were classified if met these conditions: SpO2 ≤ 93% while breathing room air and PaO2/FiO2 ≤ 300 mmHg, requiring additional O2 supply. Moderately ill patients were elderly with various symptoms including polypnea and O2 requirement.

Itolizumab was administered at 200 mg per dose intravenously. According to clinical evolution of the patient and the physician criteria, a second and even a third dose of the MAb were administered each 72 h after the first dose of itolizumab. Before the treatment, informed consent was obtained from enrolled patient.

The patients were treated in Manuel Piti Fajardo Universitary Hospital in Villa Clara, a central province in Cuba, after the approval of the Ethical Committee of the hospital and the Cuban Regulatory Agency (CECMED). Currently, the trial is recruiting patients.

### Laboratory examination

Laboratory results included blood routine, leucocyte subsets and blood biochemical parameters were collected. The total number of lymphocytes and neutrophils in peripheral blood was counted by hemocytometer.

Serum samples for the study of circulating cytokines (IL-6, IL-1 and TNFα) were obtained the day receiving the itolizumab and 48 h later.

The level of inflammatory cytokines was measured using human Quantikine ELISA Kits from R&D Systems (Minneapolis, USA): Human IL-6 Quantikine ELISA Kit (Cat# S6050). Human IL-1 beta/IL-1F2 Quantikine ELISA Kit (Cat# SLB50). Human TNF- alpha Quantikine ELISA Kit (Cat# STA00D).

### Statistical analysis

The baseline and demographic variables were described as percentages and the quantitative parameters as mean and standard deviation. Statistical significance between groups was evaluated using nonparametric Mann Whitney and Wilcoxon test. The receiver operator characteristic (ROC) curves were built to assess IL-6 predictive value of disease severity. The statistical analyses were performed with GraphPad 5. The statistical data were considered significant if *p* < 0.05.

## Data Availability

The datasets used and analyzed during the study are available from the corresponding author on reasonable request.

## Abbreviations

IL-6: Interleukin 6
MAb: Monoclonal antibody
INF-γ: Interferon gamma
TNFα: Tumour necrosis factor alpha
IgG1: Immunoglobulin G1
pMAPK: Phosphorylated mitogen-activated protein kinase
pSTAT3: Phosphorylated signal transducer and activator of transcription 3
CECMED: Centro para el control Estatal de medicamentos, equipos y dispositivos médicos
COPD: Chronic obstructive pulmonary disease
NSCLC: Non-small cell lung cancer
NLR: Neutrophil-to-lymphocyte ratio
PLR: Platelet-to-lymphocyte ratio
ALT: Alanine aminotransferase
ROC: Receiver operator characteristic
AUC: Area under curve
CRS: Cytokine release syndrome
